# Supplementary material for: Light alters the impacts of nitrogen and foliar pathogens on the performance of early successional tree seedlings
Source: PeerJ. 2021 Jul 8;9:e11587. doi: 10.7717/peerj.11587 (PMC8272923; doi:10.7717/peerj.11587)
Supplement: Supplemental Information 4 — Supplementary methods, figures, and ANOVA tables [file peerj-09-11587-s004.docx]

**Supplementary Methods**

*Effects of Fungicide on Plant Growth*

To determine whether fungicide application had any direct effects on seedling biomass, we conducted a greenhouse experiment in the absence of fungal pathogens. The experiment ran in the University of North Carolina-Chapel Hill greenhouse from September through mid-December 2014. Seedlings in this greenhouse study were handled similarly to the seedlings in the field experiment, except that all seedlings in the greenhouse study received the high nitrogen treatment—10 g N m^-2^ via five aqueous applications over ten weeks (92 mg NH_4_^+^NO_3_^-^ plant^-1^ application^-1^). We applied fungicide biweekly to half the seedlings, and left the other half untreated. We estimated foliar damage from enemies to be zero for all seedlings, and therefore any differences in biomass could be attributed to direct effects of the fungicide treatment. In mid-December, we harvested and weighed biomass in a procedure identical to the one described for the field study. Although plant height when fungicide application began did not differ by spraying treatment (P = 0.19), initial plant height predicted final biomass (P = 0.05). Thus, to account for differences in plant size, we included initial plant height as a covariate. The fungicide treatment reduced total biomass by ~ 10%, but this effect was only marginally significant (P = 0.084; Table S1a; Fig. S1a). However, environmental differences between the greenhouse and the field may account for the effect of fungicide: in the field, ultraviolet radiation degrades fungicide and rainfall washes fungicide off leaves, requiring regular application to effectively maintain the treatment. By mimicking the field application frequency in the greenhouse, we may have exposed plants in the greenhouse study to higher fungicide concentrations than plants in the field study. Furthermore, if fungicide had direct negative effects on plant performance in the field, positive effects of the fungicide treatment would indicate strong indirect positive effects of fungicide on plant performance (e.g., growth facilitated by reduced pathogen infection).

**Fig. S1** Effects of fungicide application on a) total biomass and b) proportion aboveground biomass under greenhouse conditions, calculated using restricted maximum likelihood. Error bars represent 95% confidence intervals.

**Fig. S2** Mean light availability as measured by photon incidence for the high and low light treatments, as well as the three light levels along the light transects (T1: ~ 10 m from forest edge, T2: ~ 5 m from forest edge, T3: at forest edge). The high light, T1, and T2 treatments have nearly identical light levels; T3 had a lower level of mean light availability, and the low light treatment had the lowest.


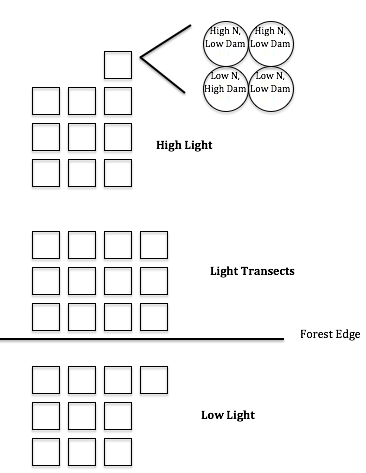


**Fig. S3** Aerial view of experimental layout. Each square represents one of 32 whole plots, while the circles show the 2 × 2 factorial subplot manipulations of nitrogen and damage treatments. Distance between each whole plot within a light treatment is about 5m, distance between high light treatment and light transects is about 20m, distance between light transects and low light treatment is about 10m.

**Table S1.** ANCOVA of effects of fungicide application and initial plant leaf number on a) total biomass and b) proportion aboveground biomass and damage treatment under greenhouse conditions, calculated using restricted maximum likelihood.

|  | **a) Total Biomass** | | | **b) Aboveground Proportion** | | |
| --- | --- | --- | --- | --- | --- | --- |
| **Predictor** | **DF** | **F value** | **P value** | **DF** | **F value** | **P value** |
| Intercept | 1 | 1232.55 | <0.001 | 1 | 1835.85 | <0.001 |
| Initial Height | 1 | 4.59 | 0.042 | 1 | -- | -- |
| Spraying | 1 | 3.24 | 0.084 | 1 | 0.54 | 0.47 |
| Error | 25 | -- | -- | 26 | -- | -- |

**Table S2.** Summary of linear mixed effects model of mean cubed-root foliar fungal damage in response to nitrogen and light availability and fungicide spraying, calculated using restricted maximum likelihood. Random effects of plant ID were nested within replicate cage. Leaves for which there were no data were omitted. Variances were allowed to differ by light treatment.

| **Predictor** | **numDF** | **denDF** | **F-value** | **p-value** |
| --- | --- | --- | --- | --- |
| Intercept | 1 | 436 | 888.74 | <0.001 |
| Nitrogen | 1 | 87 | 5.27 | 0.024 |
| Light | 1 | 30 | 34.54 | <0.001 |
| Fungicide | 1 | 87 | 12.96 | <0.001 |
| Nitrogen × Light | 1 | 87 | 4.18 | 0.04 |
| Nitrogen × Fungicide | 1 | 87 | 3.50 | 0.06 |
| Fungicide × Light | 1 | 87 | 59.63 | <0.001 |
| Nitrogen × Fungicide × Light | 1 | 87 | 4.97 | 0.03 |

**Table S3.** Summary of linear mixed effects model of mean cubed-root foliar fungal damage in response to nitrogen and light availability for plants not sprayed with fungicide, calculated using restricted maximum likelihood. Random effects of plant ID were nested within replicate cage. Leaves for which there were no data were omitted. Variances were allowed to differ by light treatment.

| **Predictor** | **numDF** | **denDF** | **F value** | **P value** |
| --- | --- | --- | --- | --- |
| Intercept | 1 | 206 | 518.57 | <0.001 |
| Nitrogen | 1 | 28 | 0.48 | 0.50 |
| Light | 1 | 29 | 2.74 | 0.11 |
| Nitrogen × Light | 1 | 28 | 7.54 | 0.01 |

**Table S4.** Summary of linear mixed effects model of cumulative height accumulation from 8 days after beginning the field experiment to the day of clipping in response to nitrogen and light availability (73 days), and damage treatment, calculated using restricted maximum likelihood. Random effect of plant ID was nested within replicate cage. Observations for which there were no data were omitted from analyses. Variances were allowed to differ by light treatment.

| **Predictor** | **numDF** | **denDF** | **F-value** | **p-value** |
| --- | --- | --- | --- | --- |
| Intercept | 1 | 90 | 1842.40 | <0.001 |
| Nitrogen | 1 | 90 | 5.41 | 0.022 |
| Fungicide | 1 | 90 | 2.19 | 0.14 |
| Light | 1 | 30 | 227.01 | <0.001 |
| Nitrogen × Fungicide | 1 | 90 | 0.01 | 0.91 |
| Nitrogen × Light | 1 | 90 | 8.52 | 0.004 |
| Fungicide × Light | 1 | 90 | 9.82 | 0.002 |
| Nitrogen × Fungicide × Light | 1 | 90 | 0.13 | 0.72 |
